# Detection of genetic incompatibilities in non-model systems using simple genetic markers: hybrid breakdown in the haplodiploid spider mite *Tetranychus evansi*

**DOI:** 10.1038/hdy.2016.103

**Published:** 2016-10-26

**Authors:** B Knegt, T Potter, N A Pearson, Y Sato, H Staudacher, B C J Schimmel, E T Kiers, M Egas

**Affiliations:** 1 Institute for Biodiversity and Ecosystem Dynamics, University of Amsterdam, Amsterdam, The Netherlands; 2 Sugadaira Montane Research Center, University of Tsukuba, Ueda, Japan; 3 Department of Ecological Science, Vrije Universiteit, Amsterdam, The Netherlands

## Abstract

When two related species interbreed, their hybrid offspring frequently suffer from reduced fitness. The genetics of hybrid incompatibility are described by the Bateson–Dobzhansky–Muller (BDM) model, where fitness is reduced by epistatic interactions between alleles of heterospecific origin. Unfortunately, most empirical evidence for the BDM model comes from a few well-studied model organisms, restricting our genetic understanding of hybrid incompatibilities to limited taxa. These systems are predominantly diploid and incompatibility is often complete, which complicates the detection of recessive allelic interactions and excludes the possibility to study viable or intermediate stages. Here, we advocate research into non-model organisms with haploid or haplodiploid reproductive systems and incomplete hybrid incompatibility because (1) dominance is absent in haploids and (2) incomplete incompatibility allows comparing affected with unaffected individuals. We describe a novel two-locus statistic specifying the frequency of individuals for which two alleles co-occur. This approach to studying BDM incompatibilities requires genotypic characterization of hybrid individuals, but not genetic mapping or genome sequencing. To illustrate our approach, we investigated genetic causes for hybrid incompatibility between differentiated lineages of the haplodiploid spider mite *Tetranychus evansi*, and show that strong, but incomplete, hybrid breakdown occurs. In addition, by comparing the genotypes of viable hybrid males and inviable hybrid male eggs for eight microsatellite loci, we show that nuclear and cytonuclear BDM interactions constitute the basis of hybrid incompatibility in this species. Our approach opens up possibilities to study BDM interactions in non-model taxa, and may give further insight into the genetic mechanisms behind hybrid incompatibility.

## Introduction

When two related species interbreed, they sometimes form hybrid offspring. Hybrids often have reduced fitness compared with their non-hybrid siblings, typically caused by either a loss in fertility, a loss in viability, or both. This hybrid incompatibility is explained by negative fitness effects of interacting loci, called Bateson–Dobzhansky–Muller (BDM) incompatibilities (Bateson, 1909; Dobzhansky, 1936; Muller, 1942, Maheshwari and Barbash, 2011). Under the BDM model, epistatic interactions among alleles of heterospecific origin result in decreased fitness. Evolutionary biologists are interested in BDM incompatibilities because understanding the underlying genetics provides insight into the evolutionary processes shaping differentiation between populations. For example, it is currently not known which evolutionary forces are the major cause of hybrid incompatibilities (Maheshwari and Barbash, 2011), what the fate is of hybrid incompatibilities upon renewed contact between previously separated populations (Lindtke and Buerkle, 2015), and to what extent populations harbour polymorphisms for BDM interactions (Cutter, 2012).

Several cases of hybrid incompatibility have been genetically investigated in *Drosophila* and other model systems (reviewed in Presgraves, 2010; Rieseberg and Blackman, 2010; Maheshwari and Barbash, 2011; Chae *et al.*, 2014). However, this reliance on model organisms is unfortunate for two reasons. First, most well-documented cases of hybrid incompatibility involve diploid species; only 3 out of the 35 studies cited in the reviews mentioned above investigate non-diploid hybrids. Diploids have an inherent disadvantage for detecting incompatible allelic interactions in hybrids, because dominant allelic interactions can mask recessive interactions. Given that both theory (Charlesworth *et al.*, 1987) and empirical work (Tao and Hartl, 2003) suggest that incompatibility alleles are mostly recessive, dominance thus complicates the detection of BDM interactions in diploids. One possible solution to the problem of dominance would be to extend crossing by one generation, that is, sampling second-generation (F2) hybrids. Recombination between parental chromosomes in the F1 generation breaks up co-adapted gene complexes and detrimental interactions are therefore more likely to be expressed in the F2. Known as ‘hybrid breakdown', F2 hybrids are generally more strongly affected by detrimental epistatic interactions than F1 hybrids (Stebbins, 1958). In many study systems, however, the parental species have diverged to an extent that hybrid breakdown is complete and no F2 can be obtained (Orr and Presgraves, 2000). Consequently, sampling F2 hybrids for the detection of BDM incompatibilities is only possible in an early stage of speciation, when hybrid incompatibility is incomplete.

The second major problem with using model organisms for detection of BDM incompatibilities is that sampling inviable individuals for genetic analysis may be difficult, because their development is often aborted at an early stage (Xu and He, 2011). With sophisticated tools, inviable individuals can sometimes still be sampled in model organisms, but without such methods researchers have to infer inviable genotypes by their absence in viable individuals (for example, Matute *et al.*, 2010), or study hybrid sterility instead. However, hybrid sterility and inviability can have different genetic causes (Xu and He, 2011), necessitating the study of both. In addition, inferring inviable genotypes from their absence in viable individuals overlooks the possibility of segregation distortion, that is, segregation of alleles deviating from expected Mendelian ratios, whereas hybrids might be especially vulnerable to unleashed segregation-distortion elements due to the breaking up of co-adapted gene complexes (Johnson, 2010).

Which systems are not vulnerable to the problems above, and are promising model systems for hybridization genetics? Ideally, research effort should be focused on (1) identifying hybrid incompatibilities that are incomplete, and (2) in sexual species with haploid life stages.

**Figure 1:**
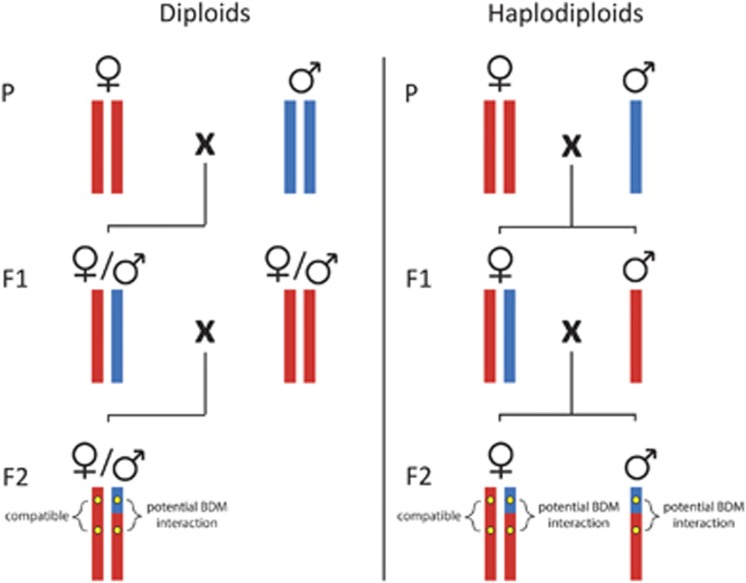
Detecting recessive Bateson–Dobzhansky–Muller (BDM) incompatibilities in diploids vs haplodiploids. In the parental generation (P) a female from one species is crossed with a male from another, related species. In diploids, the offspring generation (F1) then consists of hybrid, non-recombinant males and females. In haplodiploids, F1 females also have hybrid, non-recombinant genotypes, but F1 males have non-hybrid, ‘pure' maternally derived genotypes. When F1 females are backcrossed, diploid F2 offspring will have two haploid chromosome sets, one (maternally derived) recombinant set and one (paternally derived) non-recombinant set. In haplodiploids, the situation is the same for F2 females, but F2 males are haploid and carry only one recombinant chromosome set. If we assume that the BDM incompatibility shown in the F2 generation is recessive, then it will only be expressed in haplodiploid F2 males. Vertical bars represent haploid chromosome sets, coloured according to their species of origin. In the F2 generation only one of many theoretically possible genotypes is shown, depending on the number of recombination events and their locations in the genome.

Incomplete or partial incompatibility occurs either when not all hybrid individuals are affected, or when hybrids have reduced fitness but are still viable and fertile. For example, Hou *et al.* (2015) found that hybrid incompatibilities in the yeast *Saccharomyces cerevisiae* are condition specific, and growing them on different media allowed sampling of otherwise inviable individuals. By comparing genomes of viable and inviable individuals, these authors directly identified a two-locus BDM incompatibility, whose existence was previously disputed in yeast. The advantage of haploidy for hybridization genetics is that dominance effects are absent: any incompatibility will be expressed directly (Figure 1). Therefore, studying hybrid incompatibilities in haploid life stages does not require introgression designs or extensive hybrid F2 sampling to expose recessive deleterious interactions. In sexual eukaryotes, haploid multicellular life stages occur in a variety of organisms, including red, brown and green algae, mosses, ferns and fungi, and at least 20% of all animal species are estimated to show haplodiploid reproduction (Crozier and Pamilo, 1996). Given that most hybridization research has focused on diploids, extending the search for hybrid incompatibilities to eukaryotes with a partly haploid life cycle has the simultaneous advantage of allowing generalizations about the genetic drivers of BDM incompatibilities across a wider taxonomic diversity.

Here, we demonstrate the advantages of studying hybrid incompatibility in haploid life stages of species with incomplete incompatibility. We investigated hybrid breakdown and its genetic causes in a haplodiploid animal, the tomato red spider mite *Tetranychus evansi* Baker and Pritchard (Acari: Tetranychidae), a specialist herbivore on Solanaceae. Two genetic lineages of *T. evansi* have been described on the basis of differentiation of both nuclear and mitochondrial loci (Gotoh *et al.*, 2009). It is unknown if these genetic lineages occur sympatrically in their native range, or if they occupy different ecological niches, but interlineage hybrids have been recorded in the field (Boubou *et al.*, 2012), providing evidence that the two genetic lineages hybridize in nature. By performing controlled crosses between the genetic lineages, we show that strong but incomplete hybrid breakdown occurs. In addition, through sampling viable hybrid males and unhatched inviable hybrid male eggs, and by comparing their genotypes for eight microsatellite loci, we show that BDM incompatibilities underlie hybrid breakdown in this species. Finally, using 16S bacteriome sequencing, we show that it is unlikely that microbial endosymbionts are responsible for the observed reproductive incompatibilities.

## Materials and methods

### Mite populations and rearing conditions

*Tetranychus evansi* Algarrobo-1 was collected near Malaga, Spain, on a single *Solanum nigrum* (Solanaceae) plant in 2011 (36° 45.487' N, 4° 02.407' W). On the basis of mitochondrial *CO1* sequencing, this population was previously shown to belong to ‘lineage I' (Alba *et al.*, 2015). We adhere to lineage names as given in Boubou *et al.* (2012). *T. evansi* Viçosa-1 was collected in a glasshouse at the Federal University of Viçosa, Brazil, on *Solanum lycopersicum* cv. Santa Clara (Solanaceae) in 2002 (20° 45.473' S, 42° 52.163' W), and reared on detached leaves of the same tomato cultivar. On the basis of *CO1* sequencing, this population belongs to ‘lineage II' (Alba *et al.*, 2015). Mass cultures from both populations were established in our laboratory in Amsterdam in 2011 and 2010 respectively, and remained in culture for at least two years before the start of the experiments. *T. evansi* Viçosa-1 was exported from Brazil under export permit 03/2010/UTRA-VIÇ/DT-MG (National Plant Protection Organization of Brazil; register number BR-334), and imported into The Netherlands under declaration number 2010/016 (Plant Protection Service of The Netherlands). We maintained the cultures on detached leaves of *S. lycopersicum* cv. Castlemart plants (hereafter ‘tomato'). Tomato plants were grown in a greenhouse (25: 18 °C; 16: 8 h photoperiod; 50–60% relative humidity) for 4–6 weeks, after which leaves were detached and put flat (adaxial side up) on wet cotton wool to keep them hydrated and to prevent mites from escaping. Detached leaves with mites were kept in a climate room (25 °C; 16: 8 h light: dark photoperiod; 60% relative humidity; 300 μmol m^−2^ s^−1^ light intensity).

### Crosses

Prior to crossing, we transferred adult females from both mass cultures to fresh, detached tomato leaflets placed on wet cotton wool with their abaxial side up, 30 females per leaflet, and allowed them to oviposit for 2 days before being removed, thereby generating a cohort of offspring. After 10–12 days, the resultant offspring entered the quiescent teleiochrysalis stage, and females and males could be distinguished by their idiosomal width. As mating occurs only after adult emergence, teleiochrysalid females are thus virgin. We transferred teleiochrysalid females to fresh tomato leaflets, 25 females per leaflet, to ensure that they remained unmated. Subsequently, reciprocal crosses between, and control crosses within the two populations (in total 4 treatments) were obtained by allowing adult males from the mass cultures, 25 males per leaflet, to mate with newly emerged adult females over a period of 2 days.

### Reproductive incompatibility

To assess reproductive incompatibility between our populations, we measured oviposition rate, hatch rate and sex ratio over two generations in all four cross treatments. We placed 40 mated females of each cross (160 in total) individually on tomato leaf discs (Ø=14 mm) 3 days after adult emergence, and allowed them to oviposit. To avoid crowding in the F1 generation, we transferred each adult female to a fresh leaf disc three times (on day 2, day 4, and day 7) before removing the female (on day 9). We counted oviposition rate directly after female removal, and only included oviposition scores of females that survived until we transferred or removed them. Previous research estimated that *T. evansi* eggs hatch after an average of 4.1 (s.e.=0.09) days following oviposition, with a s.d. of 0.5 days (Bonato, 1999). Therefore, we determined the F1 hatch rate eight days after removing the adult female (that is, eight s.d. above the mean), and considered eggs inviable if they were still unhatched. Another 6 days later, that is, 2 weeks after removing the adult females, we recorded the gender of the surviving F1 individuals and transferred teleiochrysalid F1 females to fresh leaf discs. These F1 females thus remained unmated and produced only haploid male offspring. To assess whether fertilization could ‘rescue' hybrid breakdown, we also allowed virgin F1 females, 30 in each cross, to mate with adult males of either parental line in the same way as described above, thus performing backcrosses in both directions. Because adult females require on average one day to emerge from teleiochrysalis and another 2 days before they lay their first egg (Bonato, 1999), we measured F1 oviposition between 3 and 5 or between 4 and 6 days after adult emergence. We measured F2 hatch rate as described above, that is, 8 days after oviposition.

### Statistics

We used R v3.0.1 (R Development Core Team, 2013) for statistical analyses. We analyzed oviposition rate (eggs/female/day) assuming Gaussian distribution, and hatch rates and sex ratios assuming binomial distribution. In all models the variable of interest was cross (four levels), which we included as a fixed term. We included leaf disc (4 levels) and mother (4 × 40 levels) as random terms in the models for parental oviposition, F1 hatch rate and F1 sex ratio, and oviposition period (3–5 or 4–6 days) and fertilization (mated and unmated) as fixed terms in the models for F1 oviposition and F2 hatch rate. We inspected model results by plotting residuals against fitted values and against all model variables, and confirmed the absence of negative fitted values, residual patterns, and, in the case of Gaussian models, non-normality and heteroscedasticity. For binomial models we confirmed the absence of overdispersion by dividing the sum of squared residuals by the residual degrees of freedom, and considered values >1.5 to be inappropriate. We fitted mixed models using package lme4 (Bates *et al.*, 2013). We assessed the significance of cross treatments using F tests or likelihood ratio tests, while evaluating contrasts between treatments using package multcomp (Hothorn *et al.*, 2008) or, in the case of mixed binomial models, by pooling factor levels until only significant contrasts remained (Crawley, 2013).

### Sampling for genetic analysis

**Figure 2:**
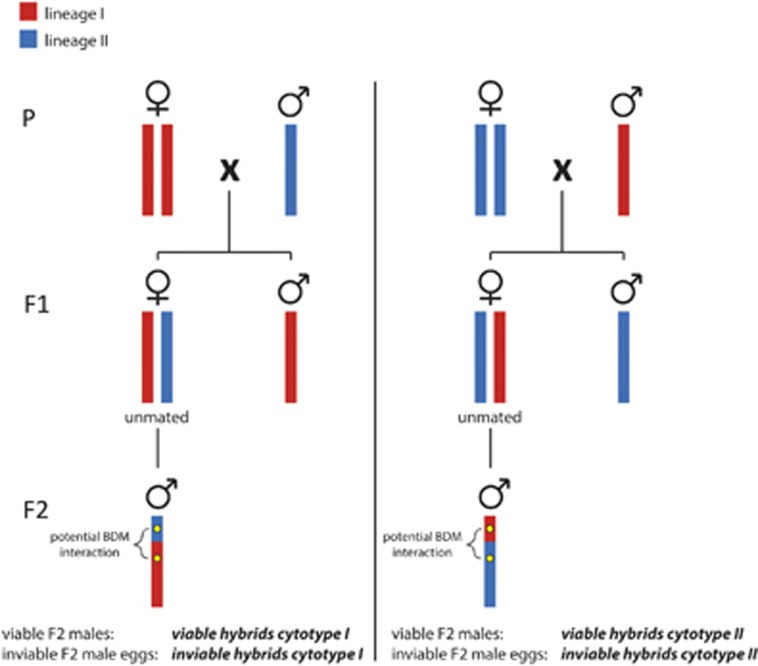
Production of recombinant F2 males via reciprocal interlineage crosses. In the parental generation (P) females from one lineage are crossed with males from the other lineage and *vice versa* (left and right panels show reciprocal crosses). Offspring (F1) consist of hybrid, non-recombinant females and non-hybrid males. F1 females are kept unmated to produce only hybrid, recombinant sons (F2). Vertical bars represent haploid chromosome sets, coloured according to their lineage of origin. In the F2 generation only one of many theoretically possible genotypes is shown, depending on the number of recombination events and their locations in the genome. The different hybrid groups from each cross that are included in the genetic analysis are indicated at the bottom of the figure.

The aim of our genetic analysis was to describe genotypic differences between viable and inviable F2 males. Therefore, we performed reciprocal interlineage crosses (two treatments, Figure 2) as described above, and allowed groups of mated females, 25 females per leaflet, to oviposit on tomato leaflets for 2 days. We transferred teleiochrysalid females of the resulting F1 generation to new leaflets, keeping them unmated, and allowed them to oviposit for 4 days. To be sure that unhatched F2 eggs were indicative of death rather than delayed development, as above, we sampled the resulting F2 individuals 8–10 days after oviposition. Thus, we sampled both adult F2 males and inviable F2 eggs, representing viable and inviable recombinant offspring of the two reciprocal crosses. We divided this data set into four groups according to their phenotype (viable or inviable) and cytotype (lineage I or lineage II): viable males with a lineage I cytotype (viable I, *n*=92), viable males with a lineage II cytotype (viable II, *n*=94), inviable males with a lineage I cytotype (inviable I, *n*=134), and inviable males with a lineage II cytotype (inviable II, *n*=134; Figure 2).

### DNA extraction

After sampling, we stored adult males individually in 96% ethanol for preservation. Prior to DNA extraction, we evaporated the ethanol, and then transferred the mites into 1.5 ml tubes containing 50 μl of 5% Chelex solution (Bio-Rad Laboratories, Hercules, CA, USA) and 3–4 zirconium beads. We homogenized samples for 20 s using a Precellys24 tissue homogenizer (Bertin Corp, Rockville, MD, USA), and added 2.5 μl of proteinase K (20 mg ml^−1^) to each sample, followed by incubation at 56 °C for 60 min, then denaturation of the proteinase at 95 °C for 8 min. We centrifuged samples at 14 000 rpm for 2 min, and stored them at -20 °C until amplification. We collected individual inviable eggs using an ethanol- and flame-sterilized pin, and crushed them in 10 μl of 5 × Phire buffer (Thermo Fisher Scientific, Waltham, MA, USA) in a PCR tube. Samples were centrifuged at 4000 rpm for 2 min and then stored at −20 °C. Prior to amplification, we pipetted the buffer-egg mix up and down to facilitate mixing of buffer and sample. To generate control samples for the PCR process, we followed the above processes without transferring a sample into the reaction medium.

### Amplification by PCR

For all samples, we amplified 16 microsatellite markers in 2 multiplex sets of 8 microsatellites per set (Boubou *et al.*, 2012), modified for use with Phire II Hot Start polymerase (Thermo Fisher Scientific): for adult samples, multiplex PCR was carried out in 10 μl reactions containing 2 μl 5 × Phire buffer, 2 μl dNTPs (1 mm each), 0.6 μl MgCl_2_ (50 mm), 0.2 μl of each forward and reverse primer (10 μm each), 0.08 μl of Phire Hot Start II DNA polymerase, 0.12 μl ddH_2_O and 2 μl of extracted DNA per sample. For egg samples, we replaced 5 × Phire buffer with ddH_2_O, and 2 μl of buffer-egg mix instead of extracted DNA. Each set of PCR reactions included a negative control: for adult samples, we used ddH_2_O instead of extracted DNA; for egg samples, we used 5 × Phire buffer instead of buffer–egg mix. Initial denaturation was at 98 °C for 30 s, then 35 cycles of denaturation at 98 °C for 10 s, annealing at 59 °C for 10 s and extension at 72 °C for 10 s. Final extension was at 72 °C for 60 s. PCR products were stored at −20 °C until analysis.

### Genotyping

For each sample, we loaded 2 μl of PCR product with 9.66 μl of HiDi formamide (Applied Biosystems, Waltham, MA, USA) and 0.34 μl of −500 LIZ size standard (Applied Biosystems) in a 96-well reaction plate, which we heated for 1 min at 96 °C. Two negative controls were run on each plate: the ABI control contained 2 μl of HiDi formamide instead of a PCR product; the PCR control contained 2 μl of control PCR product, as described previously. Plated samples were analyzed by capillary electrophoresis in a 3130 Genetic Analyzer (Applied Biosystems). We used GeneMapper 4.1 to analyze the output. In some instances we observed multiple peaks at one locus. Given that samples are haploid, this effect is most likely due to a stutter peak, that is, a very strong signal at an adjacent allele size, or other artefact of the PCR procedure. In such instances, we scored the largest peak as the allele size for that sample, but only if its peak area was at least double that of all other peaks at that locus. If alleles could not be distinguished by this rule, no allele was scored. In addition, as a cutoff for background noise, we also scored no allele if the peak area was lower than 400 units.

### Marker selection

To identify diagnostic markers, we genotyped 20 adult males of each parental population. Of the 16 loci, 8 markers (Supplementary Table S1) were selected for further use because they were fixed for different alleles in the parental populations and had consistent success of amplification. Hybrid individuals with no allele scored at more than two out of these eight markers were excluded from genetic analyses.

### Genetic analysis

As we selected microsatellite markers for which the parental populations were fixed for different alleles, haploid F2 males have one of two possible alleles at each locus. We contrasted genotypes among the hybrid groups viable I, viable II, inviable I and inviable II (Figure 2) in three comparisons (see below). The results of this analysis are unaffected by linkage, because if there is linkage between our loci, it will affect the genotypes of each hybrid group to the same extent. Consequently, linkage may affect the genotypic composition of each hybrid group individually, but any genotypic differences among the groups cannot be caused by linkage. Similarly, genotypic comparisons between hybrid groups also do not assume Mendelian segregation of alleles, because non-random segregation would affect each hybrid group identically.

### Genetic analysis: cytonuclear interactions

In order to assess cytonuclear BDM interactions, we compared viable I with viable II (comparison 1). The difference between the viable groups is their cytotype; hence, differences in allele frequency would indicate that some nuclear loci interact with the cytoplasm to affect viability. We contrasted allele frequencies using Fisher's exact tests of independence for each marker separately, and adjusted the resulting *P*-values using a sequential Bonferroni correction procedure.

### Genetic analysis: nuclear interactions

For assessing strictly nuclear BDM interactions we compared viable and inviable hybrid groups within each of the two cytotypes (comparisons 2 and 3). As BDM interactions involve interactions between at least two loci, contrasting patterns of single allele frequencies is insufficient. Instead, we calculated a two-locus statistic specifying the frequency of individuals for which two alleles co-occur. This is a novel approach to studying BDM incompatibilities, and we therefore describe its application and interpretation in detail.

The 8 markers that we studied form 28 unique marker pairs. Given that all loci have two possible alleles, for each marker pair there are four possible combinations: both alleles derived from lineage I (I, I), both alleles derived from lineage II (II, II), first allele derived from lineage I and second allele derived from lineage II (I, II) and first allele derived from lineage II and second allele derived from lineage I (II, I). Here we refer to these allele combinations as ‘haplotypes' hence, the frequency with which each allele combination occurs within a hybrid group is the haplotype count of that allele combination in that group. I, I and II, II are parental haplotypes, whereas I, II and II, I are recombinant haplotypes. Importantly, allele frequencies and haplotype counts are composed of the same data, and thus correlated. For example, if viable I has a lack of lineage I alleles at marker A compared with inviable I, then at marker pair AB it will most likely have a lack of I, I and I, II haplotypes and a surplus of II, II and II, I haplotypes. If, on the other hand, viable I also has a surplus of lineage I alleles at marker B, then the marker A bias might cancel out for I, I and II, II but be even stronger for I, II and II, I. These correlations will produce a linear relation between allele frequency and haplotype count; any non-linear effect, such as epistatic interactions between loci, will obscure the relation. Therefore, deviations of the linear relation between allele frequency and haplotype counts indicate allelic interactions between loci.

In order to explicitly assess the relation between allele frequency and haplotype count, we first tested the overall difference between viable and inviable hybrid groups across haplotypes using generalized Cochran-Mantel-Haenszel tests, while adjusting *P*-values with a sequential Bonferroni correction. Subsequently, we subjected both the allele frequencies and the haplotype counts to a contingency table analysis, calculating the adjusted residuals of each cell (Supplementary Appendices S1 and S2). Adjusted residuals are Pearson's residuals divided by the s.d. of all residuals, thus obtaining *z*-scores that follow a Gaussian distribution with zero mean and unit s.d. (Haberman, 1973). These residuals take into account both the sample size and the distribution of alleles or haplotypes for each locus or marker pair. Out of 224 adjusted haplotype residuals, one could not be computed because expected values equalled zero. In order to regress the adjusted haplotype residuals against some allele frequency metric, we defined an ‘allele indicator' statistic, which is the sum of two adjusted residual allele frequencies (Supplementary Appendix S3). Next, we defined a linear model that regresses adjusted haplotype residuals (continuous) on allele indicator (continuous) and an interaction between marker pair (28 levels) and haplotype (2 levels: parental, recombinant), and assessed significance of terms using likelihood ratio tests. For significant interactions, we evaluated *post hoc* contrasts using package phia (De Rosario-Martinez, 2015). Under the BDM model, we expect a significant interaction between marker pair and haplotype, and specifically such that viable individuals have more parental and fewer recombinant haplotypes than inviables. The opposite pattern, where viable individuals have more recombinant haplotypes and fewer parental haplotypes, is indicative of heterosis. Because this regression includes allele indicator as a term, any correlation between haplotypes due to differences in allele frequency is controlled for.

### Mite-associated bacteria

Hybrid breakdown is not only caused by genetic mechanisms, but also by microbes such as gut (Brucker and Bordenstein, 2013) or endosymbiotic bacteria (Vala *et al.*, 2000). We screened the parental Algarrobo-1 and Viçosa-1 populations for bacterial infections using two approaches. First, we assessed whether the two populations were infected with *Wolbachia*, *Cardinium*, and/or *Spiroplasma*, the most prevalent reproductive parasites among arthropods (Duron *et al.*, 2008). To achieve this, we extracted DNA of eight adult females of each population using the Chelex protocol described above, and subsequently amplified bacterial loci using specific primers (Supplementary Table S2). We included positive and negative controls for each locus, and confirmed successful DNA extraction by amplification of a 166 bp long fragment of the spider mite *β-actin* gene. We performed PCR reactions in 10 μl solutions containing 2 μl 5 × Phire Buffer (Thermo Fisher Scientific), 2 μl DNTPs (1 mm each), 0.5 μl of each primer (10 μm), 4.4 μl ddH_2_O, 0.1 μl Phire II Hot Start polymerase and 0.5 μl DNA sample. Initial denaturation was at 98 °C for 30 s, then 35 cycles of denaturation at 98 °C for 5 s, annealing at 57 °C (*Cardinium*), 52 °C (*Wolbachia* and *Spiroplasma*) or 58 °C (β-actin) for 5 s and extension at 72 °C for 10 s. Final extension was at 72 °C for 60 s. We checked amplification on 1% agarose gels using ethidium bromide staining.

### Mite-associated bacteria: 16S sequencing and analysis

Second, to assess infection with other potential reproductive parasites, we performed a 16S rDNA metagenomic survey of the bacterial community in and on the parental Algarrobo-1 and Viçosa-1 populations. We Chelex-extracted DNA from 10 adult females per population, pooled into two samples of 5 individuals each. To avoid problems in downstream processing due to pollution, we diluted each sample 20 times. Subsequently, bacterial DNA was amplified at LGC Genomics (Berlin, Germany), using universal 16S primers (Supplementary Table S2). Company guidelines were followed, with the exception that PCRs were run for 35 cycles. After purification and barcoding a 2 × 250 bp paired-end read library was constructed, which was sequenced on an Illumina MiSeq platform (Illumina, San Diego, CA, USA). We analyzed the output fastq files using Qiime software (Caporaso *et al.*, 2010a), and first joined forward and reverse reads with the join_paired_ends.py algorithm. Next, we pooled the reads from the two Viçosa-1 samples, but excluded the second Algarrobo-1 sample (see Results section), and then quality-filtered our sequences with the split_libraries_fastq.py algorithm. We applied a default base call Phred threshold of 20, allowing maximum three low-quality base calls before truncating a read, including only reads with >75% consecutive high-quality base calls, and excluding reads with ambiguous (N) base calls. Subsequently, we derived operational taxonomic units (OTUs) through a uclust screen (Edgar, 2010) on the GreenGenes database (DeSantis *et al.*, 2006) with the pick_open_reference_otus.py command and a similarity cutoff of 97%. Reads with no reference in the database were clustered *de novo*. The most abundant reads from each *de novo* cluster were aligned using PyNAST (Caporaso *et al.*, 2010b) and also included in the OTU table. We removed chimeric sequences with Chimera Slayer (Haas *et al.*, 2011) using the identify_chimeric_seqs.py command. Finally, we manually removed reads identified as chloroplasts and mitochondria, as well as global singletons.

### Mite-associated bacteria: 16S data interpretation

For reproductive parasites to have an effect on hybrid breakdown between host populations, it is assumed that hosts should be infected with different strains or species or with different densities of the same species (Vala *et al.*, 2000). We used two methods to assess this. First, we calculated the relative abundance of infection for each OTU, and listed OTUs for which the difference between Algarrobo-1 and Viçosa-1 was more than 0.5%. Second, to avoid overlooking differences for species with low densities, we also listed OTUs for which the difference in absolute read number between Algarrobo-1 and Viçosa-1 was larger than 100. For all listed OTUs, we gathered as much information as possible on the taxa in the resulting list of OTUs by searching available literature and GenBank references, and summarized this into a proposed function of each OTU within its environment. We judged the possibility of each OTU to function as a reproductive parasite in spider mites based on two criteria: if it has been found in association with arthropods, and if it has been shown to affect reproductive isolation in any species. If one of these criteria was met, we discussed the likelihood of this OTU to function as a reproductive parasite in spider mites specifically.

## Results

### Reproductive incompatibility

**Figure 3:**
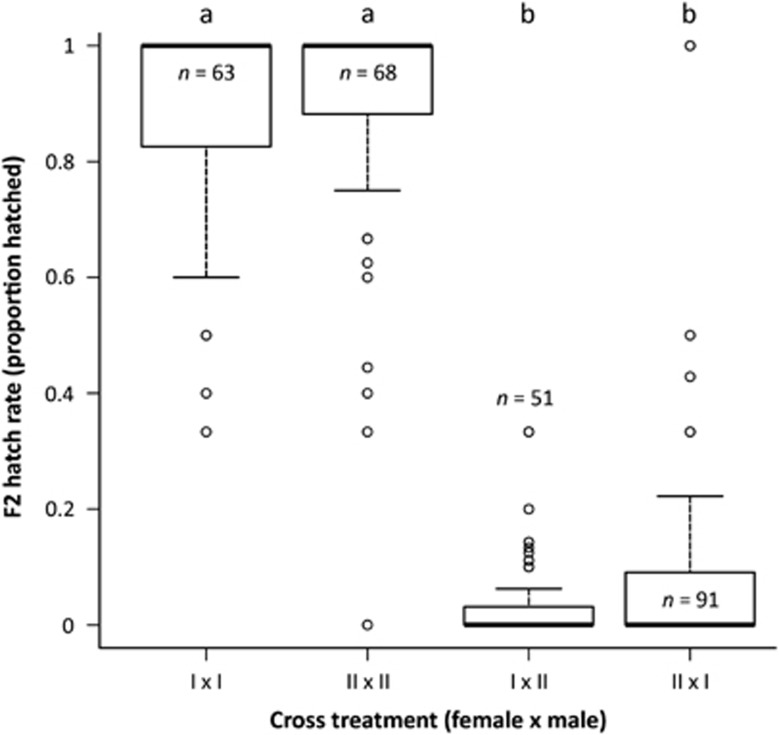
Effects of cross treatment on F2 hatch rate. Thick lines indicate treatment median; boxes encompass data from first to third quartile; whiskers indicate fences (nearest observed value ⩾first or ⩽third quartile±1.5 box height); circles indicate outliers; and different letters indicate significant differences between treatments (post hoc contrasts assessed by pooling factor levels until only significant contrasts remain, with *P*<0.05). Sample sizes are indicated within or above each box.

Parental females laid on average 4.48 (s.e.=0.13) eggs per day, irrespective of cross treatment (*χ*^2^(3)=3.04, *P*=0.39). Of these eggs, 86% hatched, also independent of treatment (*χ*^2^(3)=2.37, *P*=0.50). Typically, *T. evansi* populations consist for 20% of males (Bonato, 1999), but we found variable sex ratios among the treatments in the F1 generation, ranging from 15% males up to 36% males (*χ*^2^(3)=42.92, *P*<0.001, Supplementary Figure S1). F1 females laid fewer eggs (3.02, s.e.=0.13) than their mothers during the same part of their lives, but F1 oviposition was similar among crosses (F_3,294_=0.17, *P*=0.91). However, fertilization increased F1 oviposition by about 8% (F_1,297_=22.10, *P*<0.001). In the second generation of offspring (F2), we found strong but incomplete hybrid inviability: hatch rate of non-hybrids was ~91%, but only ~5% of the hybrid F2 eggs hatched (*χ*^2^(3)=1 679.79, *P*<0.001, Figure 3). Fertilization did not affect these hatch rates (*χ*^2^(1)=1.19, *P*=0.27). Means and sample sizes for all measurements in this section are provided in Supplementary Table S3.

### Genetic analysis

After excluding samples with more than two missing alleles, the data set contained the following samples: *n*_viable I_=88, *n*_viable II_=80, *n*_inviable I_=68, and *n*_inviable II_=60, with overall 9% missing data. Pooled across all hybrid groups, 46.0% (s.e.=1.1%) of all alleles were derived from lineage I, and the percentages varied per marker from 32% (s.e.=2.9%) to 68% (s.e.=2.8%). Within each marker pair we observed all possible haplotypes at least once, confirming that the hybrids sampled in this study have recombined genomes, and that all eight microsatellite loci are nuclear (Supplementary Table S4).

### Cytonuclear interactions

Viable I and viable II had similar allele frequencies at six microsatellite loci, ranging from 0.3 to 0.8 lineage I-derived alleles (Supplementary Figure S2). At loci F and G, however, allele frequencies differed significantly between the two viable lines, indicating that these loci, or loci linked to it, might interact with the cytoplasm and affect viability. Remarkably, the direction of difference was opposite for these two loci, with more lineage I alleles for viable I at locus G, but more lineage II alleles at locus F. This indicates that having lineage II-derived alleles in a lineage I cytotype background, and *vice versa*, can have either a positive or a negative effect on viability.

### Nuclear interactions

We found that haplotype counts across all marker pairs differed significantly between viable I and inviable I (generalized Cochran–Mantel–Haenszel test: *M*^2^=108.62, df=3, *P*<0.001) as well as between viable II and inviable II (generalized Cochran–Mantel–Haenszel test: *M*^2^=28.41, df=3, *P*<0.001). This shows that nuclear loci affect the viability of F2 hybrid males.

**Figure 4:**
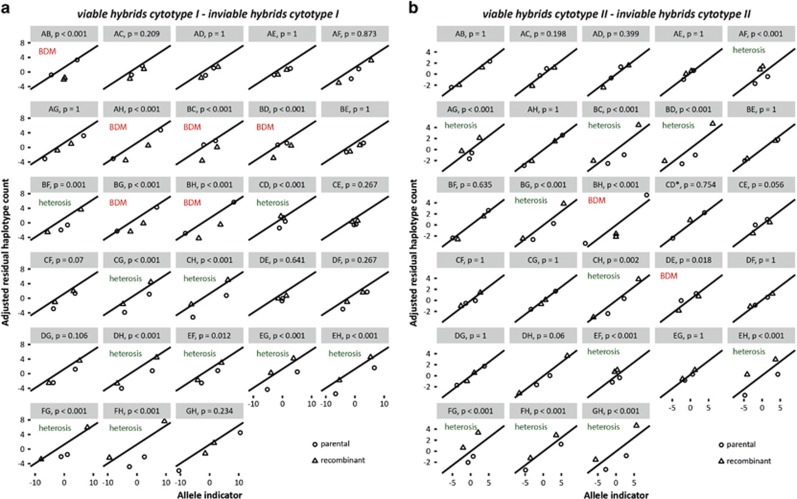
Assessment of allelic interactions at all 28 marker pairs in the viable I–inviable I comparison (**a**) and the viable II–inviable II comparison (**b**). Circles indicate parental haplotypes (I.I and II.II); triangles indicate recombinant haplotypes (I.II and II.I); and straight lines indicate predicted haplotype residuals based on allele indicator across all marker pairs. Deviations from this line suggest that allelic interactions affect haplotype residuals. The significance of the difference in deviation between parental and recombinant haplotypes is given above each panel (interaction contrasts, calculated using package phia). Whether differences indicate BDM interactions or heterosis is given in each panel. *Marker pair CD has only three values, because for the II.I haplotype expected values equalled 0, and no residual could be computed.

In order to assess whether these effects could have been caused by interactions between nuclear loci, as predicted by the BDM model, we regressed adjusted haplotype residuals on allele indicator across all marker pairs. In the absence of BDM interactions, adjusted haplotype residuals are expected to correlate linearly with allele indicator. In contrast, if BDM interactions affect the viability of individuals, then an interaction between marker pair and adjusted haplotype residuals is expected, such that viable individuals have more parental and fewer recombinant haplotypes than inviables. In the viable I–inviable I comparison, we found a significant interaction between marker pair and haplotype (F_27,55_=33.61, *P*<0.001). *Post hoc* tests indicated that 16 out of 28 marker pairs show significant differences between parental and recombinant haplotypes (Figure 4a), of which 6 conformed with the BDM model and 10 showed an opposite, heterotic pattern (Table 1). Similarly, in the viable II–inviable II comparison we also found a significant interaction between marker pair and haplotype (F_27,54_=23.08, *P*<0.001), and 13 out of 28 marker pairs were significantly different between parental and recombinant haplotypes (Figure 4b). Of these, two followed the predictions of the BDM model, and 11 marker pairs showed the opposite pattern (Table 1). Taken together, these results show that BDM incompatibilities between some loci affect viability, but recombination between other loci had positive effects.

### Reproductive parasites

We did not detect any of the known reproductive parasites *Wolbachia*, *Cardinium* or *Spiroplasma* in our *T. evansi* populations using standard PCR techniques (Supplementary Figure S3).

The 16S sequencing procedure yielded more than 10 000 reads for three of the four samples (Supplementary Table S5). One Algarrobo-1 sample, however, produced only 151 forward and reverse reads, which is unsatisfactory even for a qualitative analysis, and we therefore excluded this sample from further analysis. Joining and quality filtering reduced the remaining data set by ~30%. The uclust search against the GreenGenes database yielded 509 OTUs, of which 105 were identified as chimeric, 9 as chloroplasts and 2 as mitochondria, and another 165 were global singletons. After removing these clusters, the final data set consisted of 228 OTUs, of which 40 were clustered *de novo*. Both populations contributed equally to this final data set, with 48 955 reads from Algarrobo-1 and 47 598 from Viçosa-1.

We found 11 OTUs with more than 0.5% difference in relative abundance between Algarrobo-1 and Viçosa-1 (Table 2), and 19 OTUs for which the difference in absolute read abundance between Algarrobo-1 and Viçsoa-1 was more than 100 (Table 3). Among these OTUs we found one, *Lactococcus* sp., an uncultured glucose fermenting bacterium identified in the intestinal tract of *Apriona germari* beetle larvae (GenBank accession number EU560799.1), that was previously found to be associated with arthropods. Since bacteria belonging to this genus ferment glucose into lactic acid, it is likely that this OTU has a role in digestion rather than serve as a reproductive parasite. None of the listed OTUs has previously been associated with reproduction of a secondary species.

## Discussion

We studied hybrid incompatibility between populations of the haplodiploid spider mite *Tetranychus evansi*. Strong but incomplete hybrid breakdown was previously shown to occur between two genetic lineages of this species (Gotoh *et al.*, 2009), and we confirmed these observations with interlineage crosses between the Algarrobo-1 and Viçosa-1 populations (Figure 3). We show that the observed hybrid breakdown has a genetic component, because allele frequency patterns and two-locus haplotype counts of viable and inviable hybrids are consistently different. More specifically, we were able to show that both nuclear and cytonuclear BDM interactions lie at the basis of hybrid incompatibility in this species (Figure 4 and Supplementary Figure S2, Table 1). This is in line with previous research on haplodiploid *Nasonia* wasps, where hybrid breakdown is also explained by the combined effects of nuclear and cytonuclear defects (Gibson *et al.*, 2013). As an explanation of our results, we suggest that the two genetic lineages, over the time of their divergence, have accumulated genetic differences due to drift and adaptation, which reduce the viability of interlineage hybrids as a consequence of BDM incompatibilities.

Contrary to our expectations, we found that recombinant haplotypes at some marker pairs were overrepresented in viable genotypes compared with inviable genotypes (Figure 4, Table 1). This contrasts with the BDM model, and indicates that recombination between the two lineages also produced heterotic interactions with positive effects on viability. Heterosis is not an uncommon phenomenon, although it is usually observed in F1 progeny (Chen, 2013). Heterosis in second-generation offspring, as a counterpart to hybrid breakdown, is much less studied. In one intriguing example, Kulmuni and Pamilo (2014) demonstrated sex-specific effects of recombination between parental genomes on fitness of *Formica* wood ants, where hybrid F2 females had increased fitness but hybrid F2 males were completely inviable. Future work on hybrid breakdown in other systems could validate the simultaneous occurrence of BDM incompatibilities and heterosis, as well as the occurrence of heterosis in second- and later generation offspring.

Neither of the two parental populations was infected with *Wolbachia*, *Cardinium* or *Spiroplasma* bacteria, as assessed using standard PCR techniques (Supplementary Figure S3). In order to assess the possibility that other bacteria affected the reproductive compatibility of our mite populations, we listed the OTUs with different relative (Table 2) or absolute (Table 3) abundance between the parental populations in a 16S sequencing procedure. This analysis yielded several OTUs with different abundances, but we found no indication that these specific OTUs were associated with arthropods or functioned as reproductive parasites in any species. However, due to the short sequence length several OTUs could only be identified to the family or genus level, making it difficult to assess their role in reproductive isolation. Therefore, we cannot exclude the possibility that the microbes represented by these OTUs affect reproductive isolation in spider mites through hitherto undescribed mechanisms.

In the viable I–viable II comparison, we found significantly different allele frequencies at two loci (Supplementary Figure S2). Because all microsatellite loci used in this study are nuclear, this indicates that cytonuclear interactions affect viability. Theoretically, mitochondrial loci are expected to frequently engage in cytonuclear interactions, due to the translocation of genes for functioning of organelles to the nuclear genome (Burton *et al.*, 2013). Indeed, cytonuclear effects on hybrid breakdown have previously been found in wasps (Koevoets *et al.*, 2012). Another possibility is that epigenetically inherited differences, such as through genomic imprinting, methylation or histone modifications, cause changes in transposable element activation that are detrimental when active in a hybrid background (Lafon-Placette and Köhler, 2015). Disentangling these alternative explanations requires identification and functional characterization of the incompatibility genes followed by a gene expression assay, to see if it is the allele per se that causes the incompatibility, as would be expected for interactions between nuclear and mitochondrial loci, or if it is a dosage-dependent effect caused by deregulation of dosage-sensitive interactions.

According to the BDM model of hybrid incompatibility interactions between alleles of heterospecific origin have detrimental effects on hybrid viability. Consistent with these predictions, at some marker pairs we found an excess of parental two-locus haplotypes and a lack of recombinant haplotypes in the viable–inviable comparisons, supporting the BDM model for explaining hybrid breakdown in *T. evansi*. An open question, however, is whether BDM incompatibility is caused by many allelic interactions with small effects, or by few interactions with large effects (Maheshwari and Barbash, 2011). Although it has been shown that strong hybrid breakdown can be caused by the multiplicative effect of many loci (Dion-Côté *et al.*, 2014), genomic rearrangements such as translocations, inversions and chromosome duplications form another potential cause of incompatibility (Brown and O'Neill, 2010). Spider mites have holokinetic chromosomes, where spindle fibres attach to a diffuse kinetochore along the length of a chromosome during cell division, instead of connecting to a localized centromere. It has been suggested that due to this feature, organisms with holokinetic chromosomes exhibit aneuploidy more often, because segregation during cell division does not depend on attachment to a centromere (d'Alençon *et al.*, 2010). Thus, it is not unlikely that genome rearrangements could have a role in speciation in spider mites.

Under Mendelian segregation, we expected to find a 1:1 distribution of alleles at each locus. We observed, however, that pooled across all loci and hybrid groups only 46.0% (s.e.=1.1%) of the observed alleles derived from lineage I, and across loci there was even more variation, ranging from 32% (s.e.=2.9%) to 68% (s.e.=2.8%). Correcting for sample size within each hybrid group does not affect this pattern. Since our analysis is based on comparisons between hybrid groups, we did not assume Mendelian segregation and these biased allele distributions do not harm the validity of our results. Nevertheless, it seems that alleles do not segregate randomly, which suggests that allele distributions were affected by segregation-distortion processes such as meiotic drive or by experimental artefacts such as PCR bias (Kanagawa, 2003). Even though we tried to exclude PCR bias by excluding individuals with more than two missing alleles, it remains possible that certain inviable genotypes cease development earlier than others, and thus accumulate less DNA. If the markers of these individuals therefore have a lower chance of amplifying in PCR and also have non-random allele combinations, then that could explain the observed bias. Alternatively, since selfish genetic elements are hypothesized to have a role in BDM incompatibilities (Johnson, 2010), meiotic drive of such loci can also disturb Mendelian segregation of alleles. However, the biased recovery of alleles observed here is mild in comparison with an overall 2:1 bias in hybrid F2 *Nasonia* wasps, where cytonuclear effects and complex incompatibilities with more than two loci were suggested as potential explanations (Gadau *et al.*, 1999).

Our demonstration of BDM incompatibilities in *T. evansi* is based on comparisons between genotypes of hybrid groups, distinguished by their phenotype, viable or inviable, and their cytotype. Genotypes consist of allele frequencies at eight microsatellite loci. We employed this method, because it does not require *a priori* knowledge of the location of these markers on the genome, of linkage between them, or of possible deviations from Mendelian segregation. In addition, because we genotyped haploid males only, dominance has no role in the expression of BDM loci. These characteristics make this method suitable for investigation of hybrid incompatibilities in other non-model systems for which genetic markers are available. Although we used microsatellite markers, in principle any genetic marker can be used, including other genetic markers that are often used for non-model organisms without a sequenced genome, such as amplification fragment length polymorphisms (Vos *et al.*, 1995). Arguably, the density of genetic markers on the genome in this study is low, prohibiting any conclusions about the number and location of BDM loci in *T. evansi*. Nonetheless, we see this study as a demonstration of our method to study hybrid incompatibility in non-model organisms, and as a first step to understanding hybrid incompatibility in *T. evansi*. Future work could increase the genetic marker density in order to investigate BDM incompatibilities in *T. evansi* in more detail.

Insight into the genetic mechanisms underlying speciation requires an understanding of broad patterns across a range of taxa. However, because few studies have investigated the genetic causes of hybrid incompatibility beyond the limited set of current model organisms, several important questions remain unanswered, such as: (1) which evolutionary force predominantly drives BDM incompatibilities (Maheshwari and Barbash, 2011); (2) to what extent do hybrid inviability and sterility have different genetic causes (Xu and He, 2011); (3) what happens to BDM incompatibilities after renewed contact between previously separated populations (Lindtke and Buerkle, 2015); and (4) if it is common for populations to harbour polymorphisms for BDM interactions (Cutter, 2012). We demonstrate that by studying haploid life stages in species with incomplete incompatibility, relatively simple methods are sufficient to detect allelic interactions. We expect that a similar approach works equally well in other taxa, provided that they have haploid life stages and that both affected and unaffected stages can be sampled. Since an estimated 20% of all animals show haplodiploid reproduction (Crozier and Pamilo, 1996), there is ample opportunity for identification of other haplodiploid hybrid incompatibilities in the animal kingdom. More generally, hybrid breakdown has been reported in several taxa with haploid life stages, including ants (Kulmuni and Pamilo, 2014), wasps (Gibson *et al.*, 2013), algae (Niwa *et al.*, 2010), mosses (McDaniel *et al.*, 2008) and fungi (Turner *et al.*, 2011), indicating potentially promising species for further study. An important next step in these taxa would be to assess the feasibility of sampling inviable hybrids, which would allow direct comparisons between viable and inviable genotypes. In this respect, the importance of finding haploid systems with incomplete hybrid inviability becomes key, as they will provide valuable insight into aspects of speciation that are otherwise hard to study in these taxa.

## Data archiving

For both populations, all quality-checked and chimera-filtered 16S sequences are available at DDBJ/EMBL/GenBank under accession numbers KAHN00000000 (Algarrobo-1) and KAHO00000000 (Viçosa-1), and the versions described in this paper are the first versions KAHN01000000 and KAHO01000000. All other data sets and R scripts are available from the Dryad Digital Repository: http://dx.doi.org/10.5061/dryad.0j4m5 (Knegt *et al.*, 2016).

## Figures and Tables

**Table 1:** Number and type of interactions found across all 28 marker pairs for comparisons between viable and inviable hybrid groups

*Comparison*	*BDM interaction*	*No effect*	*Heterosis*
viable hybrids cytotype I–inviable hybrids cytotype I	6	12	10
viable hybrids cytotype II–inviable hybrids cytotype II	2	15	11

Abbreviation: BDM, Bateson–Dobzhansky–Muller.

**Table 2:** Taxonomic assignment and proposed function of OTUs with more than 0.5% difference in relative abundance between Algarrobo-1 and Viçosa-1

*Phylum*	*Class*	*Order*	*Family*	*Genus*	*Species*	*GreenGenes OTU reference*	*GenBank accession number*	*Algarrobo-1 relative abundance*	*Viçosa-1 relative abundance*	*Proposed function*
Proteobacteria	Gammaproteobacteria	Pseudomonadales	Pseudomonadaceae	*Pseudomonas*		646549	AM936014.1	31.6%	34.3%	Environmental sample form polluted industrial site
Proteobacteria	Gammaproteobacteria	Pseudomonadales	Moraxellaceae	*Psychrobacter*	*maritimus*	324143	EU000245.1	2.7%	0.0%	Bacterium sampled in South Korea, present in sea ice
Proteobacteria	Gammaproteobacteria	Enterobacteriales	Enterobacteriaceae	*Citrobacter*		559204	GU272271.1	13.1%	10.6%	Extracted from phyllosphere of dichlorvos-treated rape plants, found almost everywhere in soil and water
Proteobacteria	Gammaproteobacteria	Enterobacteriales	Enterobacteriaceae			797229	HM186527.1	2.0%	3.2%	Uncultured bacterium found at 9-52 meter below ground
Proteobacteria	Gammaproteobacteria	Enterobacteriales	Enterobacteriaceae	*Yersinia*	*ruckeri*	759061	FN668384.1	3.6%	4.7%	Fish pathogen of Atlantic salmon
Bacteroidetes	Cytophagia	Cytophagales	Cytophagaceae			637931	HM845874.1	0.0%	1.0%	Uncultured bacterium found in wounds of diabetic mice
Proteobacteria	Betaproteobacteria	Neisseriales	Neisseriaceae	*Neisseria*		4305062	JQ453291.1	0.9%	0.0%	Uncultured bacterium found in human mouth
Proteobacteria	Gammaproteobacteria	Enterobacteriales	Enterobacteriaceae			813457	HM461200.1	2.7%	2.0%	Enterobacter, cultured soil bacterium from Colombia
Proteobacteria	Alphaproteobacteria	Rhodobacterales	Rhodobacteraceae	*Paracoccus*		590586	GU061873.1	0.0%	0.6%	Uncultured marine bacterium from South Chinese Sea
Proteobacteria	Betaproteobacteria	Burkholderiales	Oxalobacteraceae	*Janthinobacterium*	*lividum*	832674	HQ538673.1	0.6%	0.0%	Soil bacterium isolated from Chinese sewer system
Firmicutes	Bacilli	Lactobacillales	Streptococcaceae	*Lactococcus*		716006	EU560799.1	0.5%	0.0%	Uncultured glucose fermenting bacterium found in intestinal tract of *Apriona germari* beetle larvae

**Table 3:** Taxonomic assignment and proposed function of OTUs with more than 100 reads difference in absolute read abundance between Algarrobo-1 and Viçosa-1

*Phylum*	*Class*	*Order*	*Family*	*Genus*	*Species*	*GreenGenes OTU reference*	*GenBank accession number*	*Algarrobo-1 number of reads*	*Viçosa-1 number of reads*	*Proposed function*
Proteobacteria	Gammaproteobacteria	Pseudomonadales	Moraxellaceae	*Psychrobacter*	*maritimus*	324143	EU000245.1	1300	0	Bacterium sampled in South Korea, present in sea ice
Bacteroidetes	Cytophagia	Cytophagales	Cytophagaceae			637931	HM845874.1	0	468	Uncultured bacterium found in wounds of diabetic mice
Proteobacteria	Betaproteobacteria	Neisseriales	Neisseriaceae	*Neisseria*		4305062	JQ453291.1	427	0	Uncultured bacterium found in human mouth
Proteobacteria	Betaproteobacteria	Burkholderiales	Oxalobacteraceae	*Janthinobacterium*	*lividum*	832674	HQ538673.1	276	0	Soil bacterium isolated from Chinese sewer system
Proteobacteria	Alphaproteobacteria	Rhodobacterales	Rhodobacteraceae	*Paracoccus*		590586	GU061873.1	0	269	Uncultured marine bacterium from South Chinese Sea
Firmicutes	Bacilli	Lactobacillales	Streptococcaceae	*Lactococcus*		716006	EU560799.1	265	0	Uncultured glucose fermenting bacterium found in intestinal tract of *Apriona germari* beetle larvae
Proteobacteria	Betaproteobacteria	Burkholderiales	Comamonadaceae	*Rhodoferax*		719367	HQ008584.1	201	0	Phototrophic bacterium sampled in freshwater in Argentina
Actinobacteria	Actinobacteria	Actinomycetales	Micrococcaceae	*Kocuria*	*palustris*	1002005	JF064759.1	185	0	Aerobic actinobacterium found on *Typha angustifolia* leaves on the Danube river
Bacteroidetes	Flavobacteriia	Flavobacteriales	Weeksellaceae	*Chryseobacterium*		656229	GQ246712.1	161	0	Found in and frequently associated with dairy products
Proteobacteria	Gammaproteobacteria	Legionellales				*De novo* cluster 43		0	160	Unknown Legionellales bacterium
Firmicutes	Bacilli	Lactobacillales	Streptococcaceae	*Streptococcus*		1082539	JF146348.1	0	155	Uncultured bacterium found at human skin
Firmicutes	Bacilli	Bacillales	Bacillaceae	*Bacillus*	*licheniformis*	574051	FJ907196.1	0	147	Found in melon juice, but usually associated with soil and ground-dwelling birds
Proteobacteria	Betaproteobacteria	Burkholderiales	Alcaligenaceae	*Achromobacter*		558264	GQ417006.1	0	145	Ubiquitous bacterium sampled on metal objects
Actinobacteria	Actinobacteria	Actinomycetales	Promicromonosporaceae	*Cellulosimicrobium*		732609	AB489904.1	0	142	*Luteimicrobium subarcticum*, sampled in soil from the subarctic Rishiri Island (Japan)
Proteobacteria	Gammaproteobacteria	Xanthomonadales	Sinobacteraceae	*Nevskia*	*ramosa*	104155	JF006394.1	0	129	Ubiquitous ammonia collecting freshwater bacterium
Proteobacteria	Gammaproteobacteria	Xanthomonadales	Sinobacteraceae			240468	EU234312.1	0	124	Uncultured bacterium found in penicillin-polluted wastewater
Proteobacteria	Gammaproteobacteria	Xanthomonadales	Xanthomonadaceae	*Stenotrophomonas*		1083508	JF037881.1	0	112	Bacterium from a diverse group of soil bacteria/pathogens
Proteobacteria	Alphaproteobacteria	Caulobacterales	Caulobacteraceae			866365	JF043115.1	111	0	Unknown Caulobacteraceae
Proteobacteria	Gammaproteobacteria	Aeromonadales	Aeromonadaceae	*Aeromonas*		839235	HM779182.1	106	0	Dominant bacterium found in zebrafish gut
