# Supplementary material for: Detection of genetic incompatibilities in non-model systems using simple genetic markers: hybrid breakdown in the haplodiploid spider mite Tetranychus evansi
Source: Heredity (Edinb). 2016 Oct 26;118(4):311–21. doi: 10.1038/hdy.2016.103 (PMC5345600; doi:10.1038/hdy.2016.103)
Supplement: Supplementary Information [file hdy2016103x1.doc]

Supporting Information Tables S1-S5, Figs S1-S3, Appendices S1-S3, supporting literature

Article title:

**Detection of genetic incompatibilities in non-model systems using simple genetic markers: hybrid breakdown in the haplodiploid spider mite *Tetranychus evansi***

Bram Knegt^1^, Tomos Potter^1^, Nigel Pearson^1^, Yukie Sato^1,3^, Heike Staudacher^1^, Bernardus C. J. Schimmel^1^, Toby Kiers^2^ and Martijn Egas^1^

^1^Institute for Biodiversity and Ecosystem Dynamics, University of Amsterdam, P.O. box 94248, 1090 GE Amsterdam, Netherlands;

^2^Department of Ecological Science, Vrije Universiteit, 1081 HV, Amsterdam, Netherlands

^3^Sugadaira Montane Research Center, University of Tsukuba, Sugadaira Kogen 1278-294, Ueda, Nagano 386-2204, Japan

Corresponding author:

Bram Knegt, email: bramknegt@gmail.com

Table S1. Specifications of microsatellite markers used in genetic analyses. Allele sizes were determined by genotyping 20 adult males of the parental populations. All eight markers were fixed for different alleles in the parental populations. Each primer pair had a fluorescent dye attached to either the forward or reverse primer indicated by colour codes *PET*, *VIC*, or *FAM*.

| **Locus name (this study)** | **Locus name (Boubou et al. 2012)** | **Allele size lineage I (bp)** | **Allele size lineage II (bp)** | **Primer sequence (5’ to 3’)** |
| --- | --- | --- | --- | --- |
| A | evaTG1-D9^SA^ | 253 | 247 | Fw: *PET*-GCCAATTGAAGGGTTACAGG  Rev: CATTCACAAGCAATGTTATTACCAG |
| B | evaTC2-A2 | 170 | 172 | Fw: *PET*-CAATTGATGGTTTCTGTTTGG  Rev: CATTATCGCTTCACTCATCGTC |
| C | evaATCT2-G11 | 121 | 124 | Fw: GGTTGACCGTGAAGAGAG  Rev: *VIC*-CAGAATGACAGTTACAATTGC |
| D | evaTC3-D3 | 121 | 124 | Fw: *FAM*-GTCGTCCGGGATTTTTTCTG  Rev: GTTGCTTGTTTAATTGTTGTCACTG |
| E | evaTC5-E6^1^ | 302 | 298 | Fw: CCACGACCAGTCTTGATTG  Rev: *PET*-GTGATGTCGAATGAGCAGG |
| F | evaTC1-H4 | 166 | 184 | Fw: *PET*-CGATATAATTGTCAATGGTG  Rev: GTATCAAGTATATTCCTATATGATG |
| G | evaATCT1-H4 | 166 | 184 | Fw: GCACAGTTGAGAACGGGCTAAG  Rev: *VIC*-CCTGTTTCTTTACTCATCCTGTCCC |
| H | evaTC1-A12 | 166 | 183 | Fw: AGCATTTTAATGTTTCCTTTG  Rev: *FAM*-GTTTCACTTGTAAATGGCTATG |

Table S2. Specifications of primers used for the detection of symbionts.

| **Target** | **Primer** | **Sequence** | **Locus** | **Fragment size** | **Reference** |
| --- | --- | --- | --- | --- | --- |
| *Wolbachia* | 76F | 5'-TTGTAGCCTGCTATGGTATAACT-3' | *16S* | 900 bp | O'Neill et al. 1992 |
|  | 1012R | 5'-GAATAGGTATGATTTTCATGT-3' |  |  |  |
| *Cardinium* | CLO-F | 5'-GCGGTGTAAAATGAGCGTG-3' | *16S* | 450 bp | Weeks et al. 2003 |
|  | CLO-R1 | 5'-ACCTMTTCTTAACTCAAGCCT-3' |  |  |  |
| *Spiroplasma* | ApDnaAF1 | 5'-ATTCTTCAGTAAAAATGCTTGGA-3' | *dnaA* | 385 bp | Fukatsu et al. 2001 |
|  | ApDnaAR1 | 5'-ACACATTTACTTCATGCTATTGA-3' |  |  |  |
| *Tetranychus evansi* | β-actin-F | 5’-CAGCCATGTATGTTGCCATC-3’ | β*-actin* | 166 bp | Feng et al. 2010 |
|  | β-actin-R | 5’-AAATCACGACCAGCCAAATC-3’ |  |  |  |
| Bacterial DNA | 341F | 5’-TCCTACGGGNGGCWGCAG-3’ | *16S* | 444 bp | modified from Klindworth et al. 2013 |
|  | 785R | 5’-TGACTACHVGGGTATCTAAKCC-3’ |  |  |  |

Table S3. Means (standard error) and sample sizes for parental oviposition (eggs per day), F1 hatch rate (proportion hatched), F1 sex ratio (proportion male), F1 oviposition (eggs per day) and F2 hatch rate (proportion hatched) in all cross treatments (female x male). For parental oviposition, F1 hatch rate and F1 sex ratio we report a time series over four consecutive leaf discs from day 0 until day 9. Sample sizes decrease over time as data are excluded due to female mortality. Note that data from one individual female can be included in up to four time points. Hence, these data are not independent, and we included female identity as a random factor in our statistical analyses. In addition, because female mortality differed among treatments, we included the leaf disc sequence as a random factor as well.

| **Parental oviposition** | **Day 0 - 2** | ***n*** | **Day 2 - 4** | ***n*** | **Day 4 - 7** | ***n*** | **Day 7 - 9** | ***n*** |
| --- | --- | --- | --- | --- | --- | --- | --- | --- |
| I x I | 4.58 (0.38) | 33 | 5.11 (0.53) | 28 | 4.63 (0.68) | 18 | 3.18 (0.74) | 11 |
| II x II | 4.93 (0.41) | 37 | 4.68 (0.41) | 31 | 2.27 (0.60) | 17 | 3.17 (1.74) | 3 |
| I x II | 5.62 (0.40) | 38 | 5.23 (0.39) | 28 | 2.79 (0.66) | 16 | 5.50 (1.50) | 2 |
| II x I | 4.36 (0.26) | 39 | 4.93 (0.47) | 27 | 2.83 (0.44) | 23 | 4.67 (0.64) | 6 |
|  |  |  |  |  |  |  |  |  |
| **F1 hatch rate** | **Day 0 - 2** | ***n*** | **Day 2 - 4** | ***n*** | **Day 4 - 7** | ***n*** | **Day 7 - 9** | ***n*** |
| I x I | 0.85 (0.03) | 32 | 0.80 (0.04) | 28 | 0.87 (0.02) | 24 | 0.74 (0.09) | 12 |
| II x II | 0.92 (0.03) | 35 | 0.89 (0.02) | 32 | 0.85 (0.06) | 22 | 0.71 (0.16) | 7 |
| I x II | 0.92 (0.02) | 37 | 0.79 (0.05) | 34 | 0.79 (0.07) | 24 | 0.81 (0.07) | 8 |
| II x I | 0.76 (0.04) | 37 | 0.83 (0.04) | 33 | 0.85 (0.03) | 22 | 0.80 (0.05) | 9 |
|  |  |  |  |  |  |  |  |  |
| **F1 sex ratio** | **Day 0 - 2** | ***n*** | **Day 2 - 4** | ***n*** | **Day 4 - 7** | ***n*** | **Day 7 - 9** | ***n*** |
| I x I | 0.15 (0.04) | 32 | 0.13 (0.03) | 25 | 0.21 (0.04) | 24 | 0.18 (0.14) | 7 |
| II x II | 0.28 (0.05) | 34 | 0.17 (0.02) | 30 | 0.24 (0.09) | 11 | 0.39 (0.20) | 3 |
| I x II | 0.44 (0.06) | 32 | 0.32 (0.06) | 28 | 0 (0) | 6 | 0.83 (0.11) | 5 |
| II x I | 0.24 (0.06) | 29 | 0.19 (0.04) | 29 | 0.13 (0.03) | 18 | 0.28 (0.16) | 6 |
|  |  |  |  |  |  |  |  |  |
| **F1 oviposition** | **unmated** | ***n*** | **mated** | ***n*** |  | | | |
| I x I | 3.62 (0.38) | 39 | 3.31 (0.42) | 27 |
| II x II | 2.88 (0.26) | 49 | 3.41 (0.37) | 22 |
| I x II | 3.11 (0.41) | 27 | 3.34 (0.42) | 25 |
| II x I | 2.60 (0.29) | 84 | 2.69 (0.37) | 27 |
|  |  |  |  |  |
| **F2 hatch rate** | **unmated** | ***n*** | **mated** | ***n*** |
| I x I | 0.89 (0.03) | 36 | 0.92 (0.03) | 27 |
| II x II | 0.89 (0.03) | 46 | 0.93 (0.05) | 22 |
| I x II | 0.02 (0.01) | 28 | 0.06 (0.02) | 23 |
| II x I | 0.07 (0.02) | 67 | 0.05 (0.02) | 24 |

|  | ***VIABLE HYBRIDS CYTOTYPE I*** | | | | | ***VIABLE HYBRIDS CYTOTYPE II*** | | | | | ***INVIABLE HYBRIDS CYTOTYPE I*** | | | | | ***INVIABLE HYBRIDS CYTOTYPE II*** | | | | |
| --- | --- | --- | --- | --- | --- | --- | --- | --- | --- | --- | --- | --- | --- | --- | --- | --- | --- | --- | --- | --- |
| **Marker pair** | *I,I* | *II,II* | *I,II* | *II,I* | *n* | *I,I* | *II,II* | *I,II* | *II,I* | *n* | *I,I* | *II,II* | *I,II* | *II,I* | *n* | *I,I* | *II,II* | *I,II* | *II,I* | *n* |
| *AB* | 42 | 13 | 4 | 17 | *76* | 27 | 13 | 4 | 27 | *71* | 12 | 11 | 8 | 17 | *48* | 7 | 16 | 7 | 11 | *41* |
| *AC* | 16 | 21 | 23 | 6 | *66* | 11 | 28 | 19 | 10 | *68* | 7 | 22 | 16 | 12 | *57* | 8 | 14 | 8 | 14 | *44* |
| *AD* | 14 | 19 | 20 | 8 | *61* | 10 | 25 | 19 | 10 | *64* | 7 | 19 | 10 | 12 | *48* | 8 | 10 | 6 | 14 | *38* |
| *AE* | 13 | 21 | 32 | 11 | *77* | 6 | 19 | 17 | 13 | *55* | 5 | 16 | 17 | 9 | *47* | 3 | 19 | 11 | 10 | *43* |
| *AF* | 8 | 22 | 36 | 9 | *75* | 10 | 17 | 18 | 20 | *65* | 12 | 12 | 11 | 18 | *53* | 8 | 18 | 7 | 10 | *43* |
| *AG* | 28 | 15 | 18 | 17 | *78* | 5 | 23 | 24 | 12 | *64* | 6 | 24 | 16 | 8 | *54* | 8 | 18 | 8 | 9 | *43* |
| *AH* | 40 | 7 | 6 | 25 | *78* | 25 | 8 | 6 | 32 | *71* | 6 | 16 | 17 | 15 | *54* | 6 | 15 | 10 | 14 | *45* |
| *BC* | 21 | 16 | 35 | 1 | *73* | 13 | 10 | 41 | 7 | *71* | 9 | 10 | 28 | 12 | *59* | 13 | 17 | 9 | 12 | *51* |
| *BD* | 19 | 13 | 31 | 4 | *67* | 13 | 9 | 37 | 7 | *66* | 9 | 7 | 20 | 12 | *48* | 13 | 16 | 6 | 12 | *47* |
| *BE* | 20 | 12 | 46 | 5 | *83* | 15 | 10 | 26 | 6 | *57* | 8 | 12 | 26 | 6 | *52* | 6 | 17 | 15 | 11 | *49* |
| *BF* | 14 | 12 | 52 | 4 | *82* | 25 | 8 | 28 | 7 | *68* | 17 | 10 | 17 | 10 | *54* | 7 | 14 | 13 | 14 | *48* |
| *BG* | 44 | 10 | 24 | 7 | *85* | 12 | 9 | 40 | 6 | *67* | 9 | 15 | 27 | 5 | *56* | 8 | 17 | 12 | 13 | *50* |
| *BH* | 58 | 3 | 10 | 14 | *85* | 49 | 5 | 9 | 11 | *74* | 11 | 10 | 24 | 11 | *56* | 9 | 15 | 12 | 16 | *52* |
| *CD* | 20 | 44 | 2 | 4 | *70* | 21 | 47 | 1 | 0 | *69* | 22 | 32 | 0 | 0 | *54* | 27 | 25 | 0 | 0 | *52* |
| *CE* | 5 | 35 | 17 | 17 | *74* | 8 | 22 | 11 | 12 | *53* | 5 | 30 | 12 | 11 | *58* | 8 | 17 | 18 | 10 | *53* |
| *CF* | 4 | 35 | 19 | 15 | *73* | 11 | 23 | 10 | 21 | *65* | 14 | 23 | 8 | 18 | *63* | 11 | 19 | 12 | 11 | *53* |
| *CG* | 16 | 19 | 7 | 34 | *76* | 7 | 33 | 12 | 12 | *64* | 9 | 37 | 12 | 7 | *65* | 12 | 20 | 13 | 10 | *55* |
| *CH* | 18 | 6 | 5 | 47 | *76* | 18 | 9 | 4 | 40 | *71* | 12 | 30 | 10 | 13 | *65* | 13 | 17 | 14 | 13 | *57* |
| *DE* | 5 | 29 | 18 | 16 | *68* | 8 | 19 | 10 | 11 | *48* | 5 | 19 | 12 | 8 | *44* | 8 | 13 | 18 | 8 | *47* |
| *DF* | 5 | 32 | 19 | 11 | *67* | 10 | 21 | 10 | 19 | *60* | 14 | 16 | 8 | 12 | *50* | 11 | 14 | 12 | 10 | *47* |
| *DG* | 19 | 19 | 5 | 27 | *70* | 7 | 29 | 11 | 12 | *59* | 9 | 25 | 12 | 5 | *51* | 12 | 16 | 13 | 8 | *49* |
| *DH* | 21 | 7 | 3 | 39 | *70* | 17 | 9 | 4 | 36 | *66* | 12 | 21 | 10 | 8 | *51* | 13 | 13 | 14 | 11 | *51* |
| *EF* | 5 | 43 | 21 | 14 | *83* | 9 | 19 | 10 | 15 | *53* | 11 | 24 | 3 | 16 | *54* | 10 | 24 | 7 | 10 | *51* |
| *EG* | 18 | 23 | 10 | 35 | *86* | 8 | 28 | 10 | 7 | *53* | 10 | 36 | 6 | 5 | *57* | 11 | 26 | 6 | 9 | *52* |
| *EH* | 26 | 11 | 2 | 47 | *86* | 15 | 8 | 6 | 28 | *57* | 10 | 31 | 5 | 9 | *55* | 13 | 24 | 5 | 12 | *54* |
| *FG* | 14 | 28 | 6 | 37 | *85* | 13 | 29 | 17 | 4 | *63* | 16 | 30 | 14 | 0 | *60* | 20 | 29 | 2 | 2 | *53* |
| *FH* | 16 | 9 | 4 | 56 | *85* | 33 | 13 | 1 | 21 | *68* | 21 | 28 | 10 | 2 | *61* | 20 | 26 | 3 | 5 | *54* |
| *GH* | 54 | 13 | 0 | 21 | *88* | 20 | 14 | 0 | 33 | *67* | 15 | 38 | 1 | 8 | *62* | 21 | 28 | 2 | 6 | *57* |

Table S4. Haplotype counts for all hybrid groups and marker pairs. Per hybrid group, sample size is indicated in italics in a separate column.

Table S5. Number of sequencing reads after joining paired-end reads and quality filtering. As the number of forward and reverse unjoined reads is identical, this table gives the number of forward *or* reverse unjoined reads, but not their sum. For downstream analysis the Viçosa-1 samples were pooled, whereas the Algarrobo-1_2 sample was excluded.

| **Sample** | **Number of unjoined reads** | **Number of joined reads** | **Number of reads after quality filtering** | **Number of reads after OTU filtering** |
| --- | --- | --- | --- | --- |
| Algarrobo-1_1 | 77116 | 59367 | 53929 | 48955 |
| Algarrobo-1_2 | 151 | *excluded* | | |
| Viçosa-1_1 | 54915 | 42462 | 38537 | 47598 |
| Viçosa-1_2 | 14396 | 11420 | 10226 |


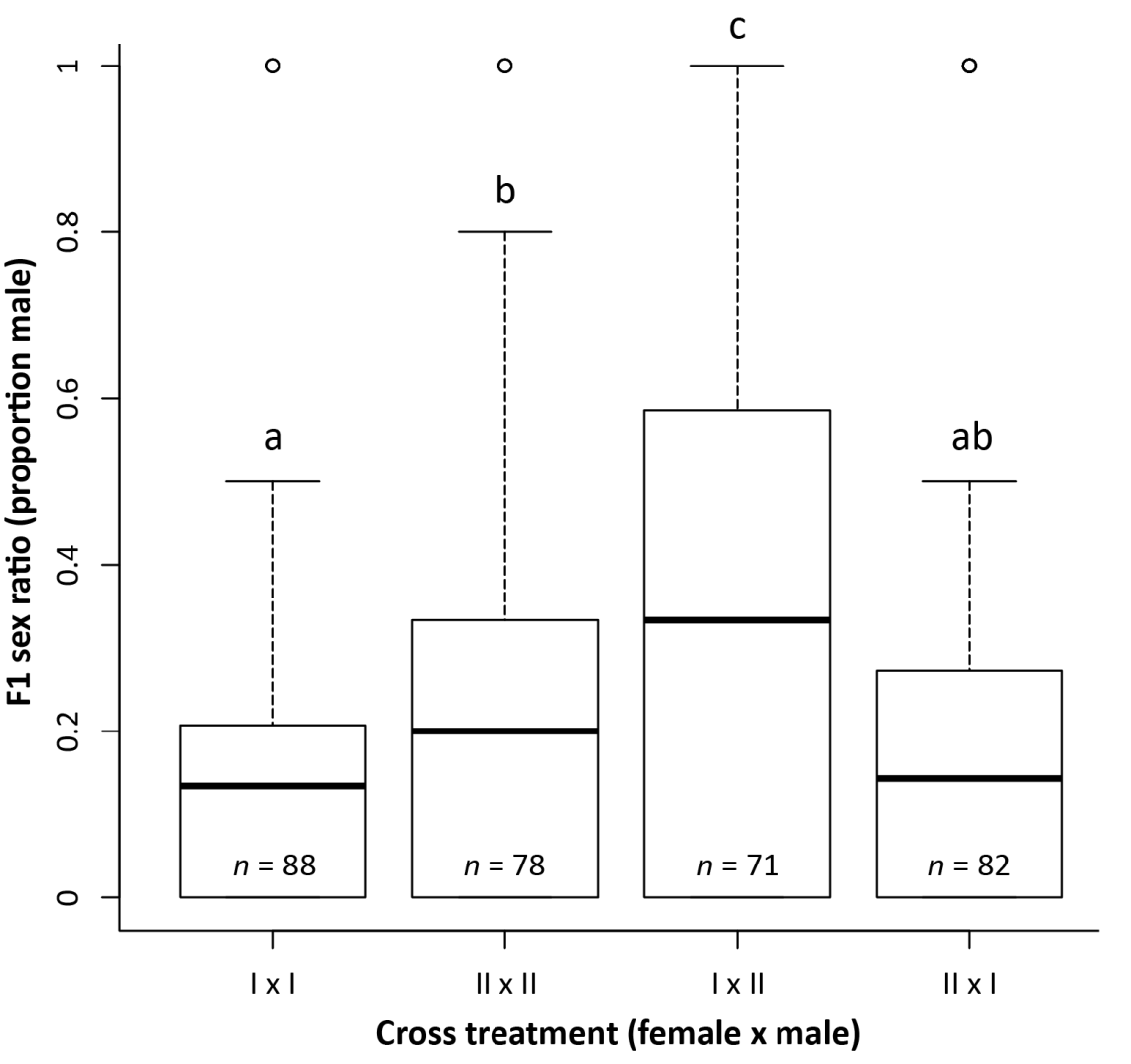


Figure S1. Effects of cross treatments on F1 sex ratio. Thick lines indicate treatment median, boxes encompass data from first to third quartile, whiskers indicate upper fence (nearest observed value ≤ third quartile + 1.5 box height), circles indicate outliers and different letters indicate significant differences between treatments (post hoc contrasts assessed by pooling factor levels until only significant contrasts remain, with p < 0.05). Sample sizes are indicated within each box.


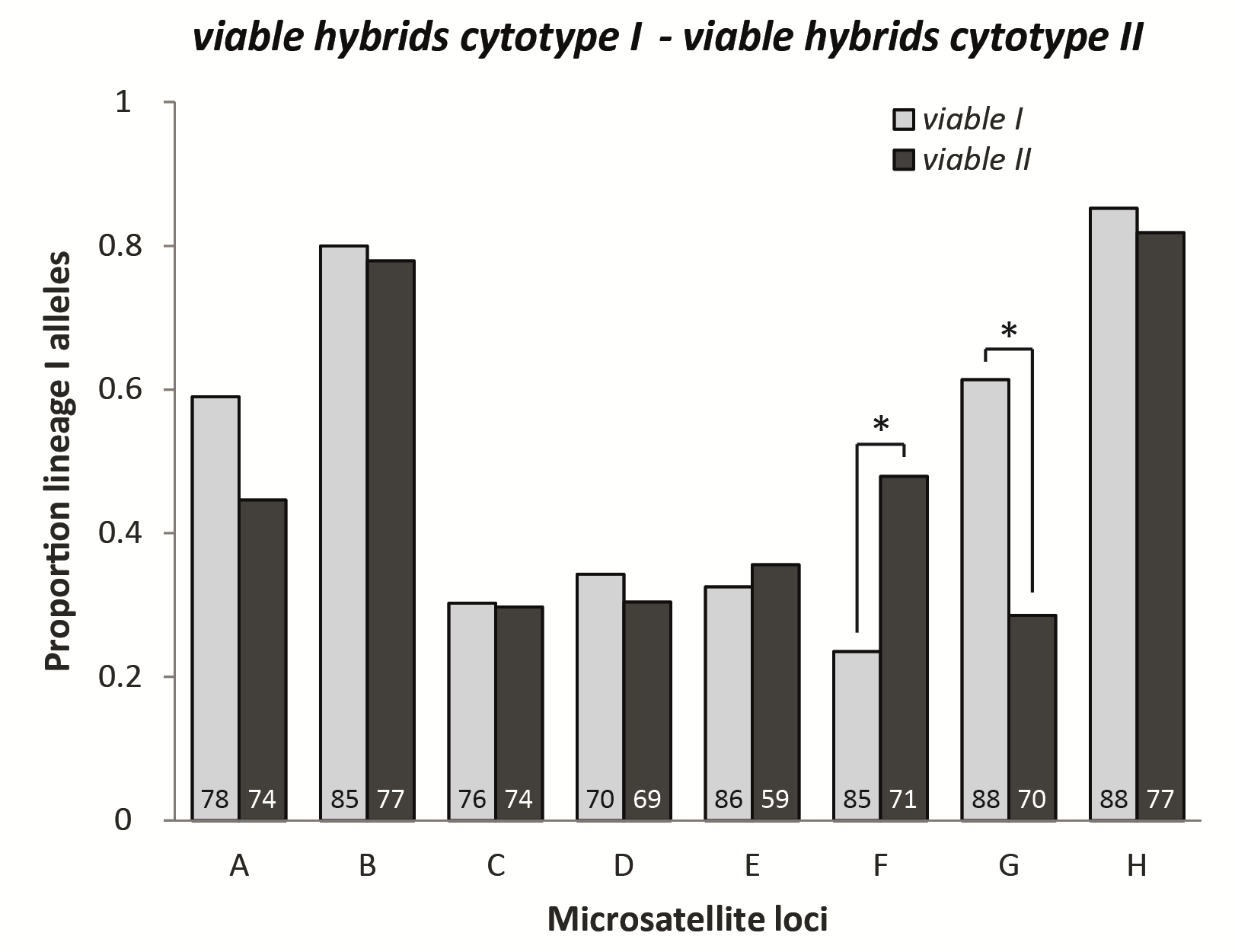


Figure S2. Microsatellite allele frequencies of the two viable groups. At each locus, the proportion of lineage I alleles is shown. Asterisks indicate significantly different allele frequencies between the two hybrid groups (Fisher’s exact test of independence, Bonferroni corrected p < 0.05). Sample sizes are indicated at the bottom of each bar.


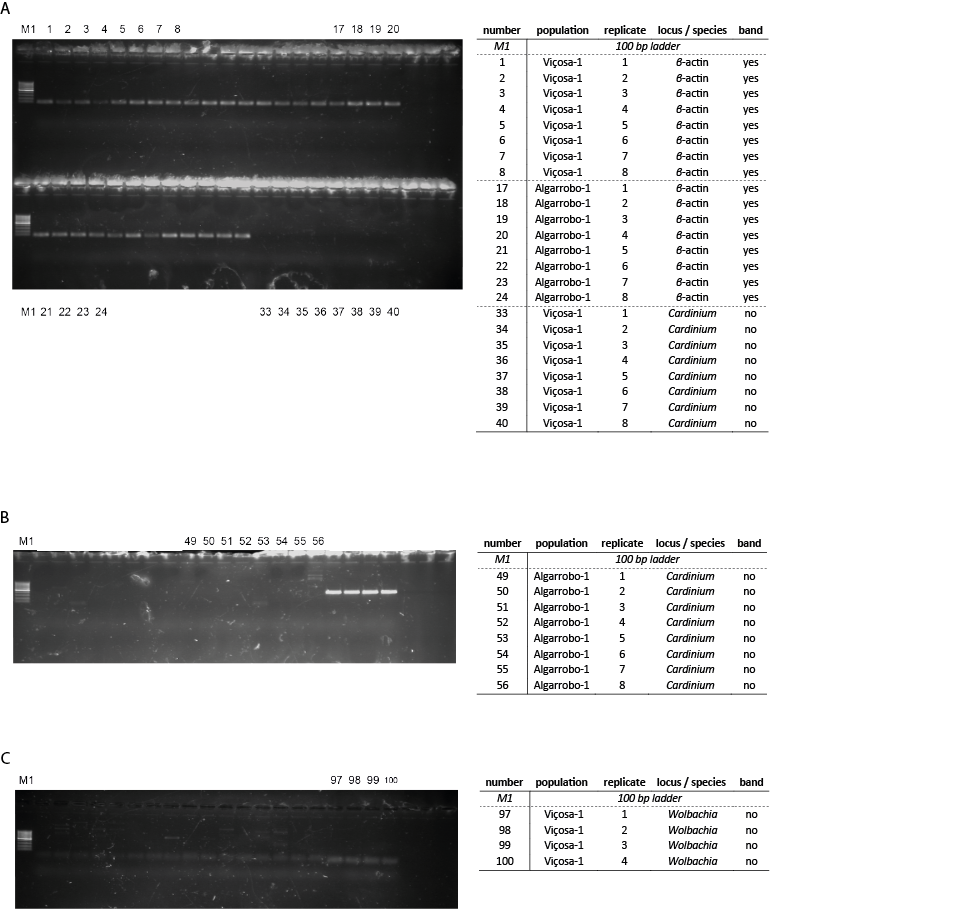


*-- figure continues on next page --*


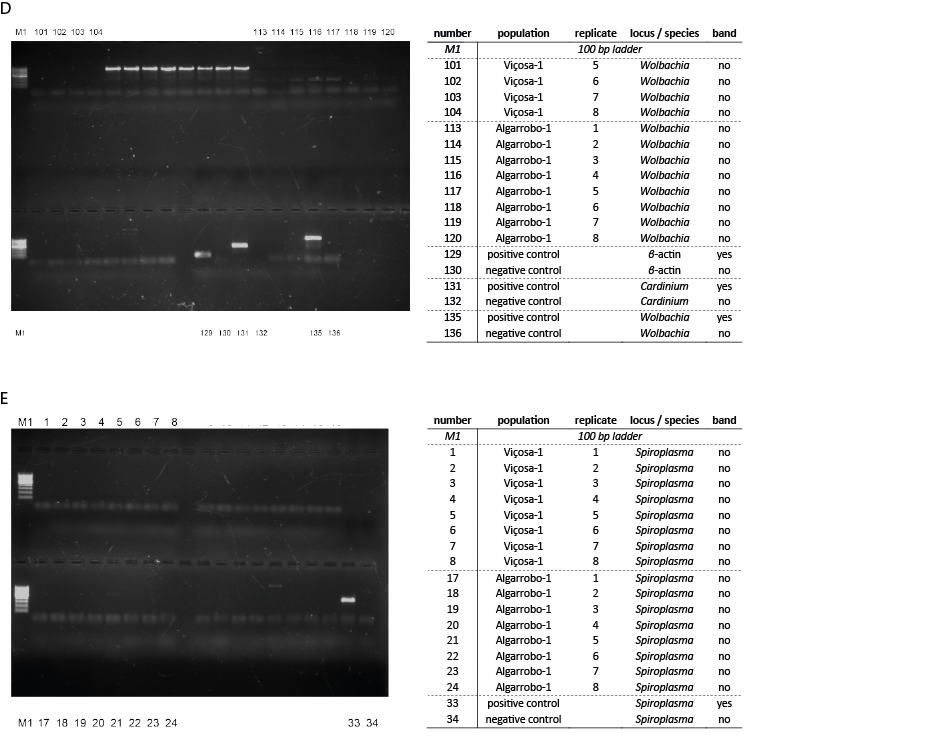


Figure S3. Endosymbiont screening results using standard PCR. PCR products were loaded on 1% agarose gels and stained using ethidium bromide. Each photo (A-E) is a separate gel. Sample specifications are given in the tables next to the gel photos, with corresponding numbers above and below the gel. Because we ran these samples within the context of another experiment, some samples are irrelevant for this research and hence excluded from the tables and not represented with a number above or below the gel.

**Appendix S1. Adjusted residual allele frequency**

This appendix illustrates the calculation of adjusted residual allele frequencies, using locus A as an example. At locus A, we observed the following alleles in *viable I* and *inviable I*:

|  | **viable hybrids cytotype I** | **inviable hybrids cytotype I** | ***sum*** |
| --- | --- | --- | --- |
| **allele type I** | 46 | 23 | ***69*** |
| **allele type II** | 32 | 34 | ***66*** |
| ***sum*** | ***78*** | ***57*** | ***135*** |

Let$O(x_{i,j})$be the number of alleles of type *i* observed in hybrid group *j*. Expected values are then obtained by: $E(x_{i,j})=\frac{\sum_{j=1}^{2} x_{i,j}\cdot\sum_{i=1}^{2} x_{i,j}}{\sum x_{i,j}}$, giving $\begin{matrix} E(x_{1,1})=\frac{69\cdot78}{135}=39.87, E(x_{1,2})=\frac{69\cdot57}{135}=29.13 \\ E(x_{2,1})=\frac{66\cdot78}{135}=38.13, E(x_{2,2})=\frac{66\cdot57}{135}=27.87 \end{matrix}$

Adjusted residuals are then given by , rendering

$$\begin{matrix} adjuste d residual (x_{1,1})=\frac{46-39.87}{\sqrt{39.87\cdot(1-\frac{69}{135})\cdot(1-\frac{78}{135})}}=2.14, adjuste d residual (x_{1,2})=\frac{23-29.13}{\sqrt{29.13\cdot(1-\frac{69}{135})\cdot(1-\frac{57}{135})}}=-2.14 \\ adjuste d residual (x_{2,1})=\frac{32-38.13}{\sqrt{38.13\cdot(1-\frac{66}{135})\cdot(1-\frac{78}{135})}}=-2.14, adjuste d residual (x_{2,2})=\frac{34-27.87}{\sqrt{27.87\cdot(1-\frac{66}{135})\cdot(1-\frac{57}{135})}}=2.14 \end{matrix}$$

Note that adjusted residuals *x_i,1_* and *x_i,2_* are each other’s additive inverses, because comparing *viable I* to *inviable I* is equivalent to comparing *inviable I* to *viable I*. Similarly, adjusted residuals *x_1,j_* and *x_2,j_* are each other’s additive inverses, because comparing allele type I to allele type II is equivalent to comparing allele type II to allele type I.

**Appendix S2. Adjusted residual haplotype count**

This appendix illustrates the calculation of adjusted residual haplotype counts, using marker pair AB as an example. At marker pair AB, we observed the following haplotypes in *viable I* and *inviable I*:

|  | **viable hybrids cytotype I** | **inviable hybrids cytotype I** | ***sum*** |
| --- | --- | --- | --- |
| **haplotype *I.I*** | 42 | 12 | ***54*** |
| **haplotype *II.II*** | 13 | 11 | ***24*** |
| **haplotype *I.II*** | 4 | 8 | ***12*** |
| **haplotype *II.I*** | 17 | 17 | ***34*** |
| ***sum*** | ***76*** | ***48*** | ***124*** |

Let$O(x_{i,j})$be the number of haplotypes of type *i* that we observed in hybrid group *j*. Expected values are then obtained by:

$E(x_{i,j})=\frac{\sum_{j=1}^{2} x_{i,j}\cdot\sum_{i=1}^{4} x_{i,j}}{\sum x_{i,j}}$, giving $\begin{matrix} E(x_{1,1})=\frac{54\cdot76}{124}=33.10, E(x_{1,2})=\frac{54\cdot48}{124}=20.90 \\ E(x_{2,1})=\frac{24\cdot76}{124}=14.71, E(x_{2,2})=\frac{24\cdot48}{124}=9.29 \\ E(x_{3,1})=\frac{12\cdot76}{124}=7.35, E(x_{3,2})=\frac{12\cdot48}{124}=4.65 \\ E(x_{4,1})=\frac{34\cdot76}{124}=20.84, E(x_{4,2})=\frac{34\cdot48}{124}=13.16 \end{matrix}$

Adjusted residuals are then given by, rendering

$\begin{matrix} adjuste d residual (x_{1,1})=\frac{42-33.10}{\sqrt{33.10\cdot(1-\frac{54}{124})\cdot(1-\frac{76}{124})}}=3.31, adjuste d residual (x_{1,2})=\frac{12-20.90}{\sqrt{20.90\cdot(1-\frac{54}{124})\cdot(1-\frac{48}{124})}}=-3.31 \\ adjuste d residual (x_{2,1})=\frac{13-14.71}{\sqrt{14.71\cdot(1-\frac{24}{124})\cdot(1-\frac{76}{124})}}=-0.80, adjuste d residual (x_{2,2})=\frac{11-9.29}{\sqrt{9.29\cdot(1-\frac{24}{124})\cdot(1-\frac{48}{124})}}=0.80 \\ adjuste d residual (x_{3,1})=\frac{4-7.35}{\sqrt{7.35\cdot(1-\frac{12}{124})\cdot(1-\frac{76}{124})}}=-2.09, adjuste d residual (x_{3,2})=\frac{8-4.65}{\sqrt{4.65\cdot(1-\frac{12}{124})\cdot(1-\frac{48}{124})}}=2.09 \\ adjuste d residual (x_{4,1})=\frac{17-20.84}{\sqrt{20.84\cdot(1-\frac{34}{124})\cdot(1-\frac{76}{124})}}=-1.59, adjuste d residual (x_{4,2})=\frac{17-13.16}{\sqrt{13.16\cdot(1-\frac{34}{124})\cdot(1-\frac{48}{124})}}=1.59 \end{matrix}$Note that adjusted residuals *x_i,1_* and *x_i,2_* are each other’s additive inverses, because comparing *viable I* to *inviable I* is equivalent to comparing *inviable I* to *viable I*.

**Appendix S3. Allele indicator statistic**

In order to regress the adjusted haplotype residuals against some allele frequency metric, we defined an “allele indicator” statistic which is the sum of two adjusted residual allele frequencies. As an example, we calculate the allele indicator of marker pair AB in the comparison of hybrid groups *viable I* to *inviable I*, and show how it is used in combination with the adjusted residual haplotype counts of marker pair AB.

At loci A and B, we obtained the following adjusted residual allele frequencies:

|  |  | **viable hybrids cytotype I** | **inviable hybrids cytotype I** |
| --- | --- | --- | --- |
| **Marker A** | **allele type I** | 2.14 | -2.14 |
|  | **allele type II** | -2.14 | 2.14 |
| **Marker B** | **allele type I** | 2.30 | -2.30 |
|  | **allele type II** | -2.30 | 2.30 |

Since the allele indicator is defined as the sum of the two respective adjusted residual allele frequencies, we obtain the following allele indicators for marker pair AB:

|  | **formula** | **calculation** | **allele indicator** | **adjusted residual haplotype count** |
| --- | --- | --- | --- | --- |
| **haplotype *I.I*** | *res_A_(x_1,1_) + res_B_(x_1,1_)* | 2.14 + 2.30 | 4.43 | 3.31 |
| **haplotype *II.II*** | *res_A_(x_1,2_) + res_B_(x_1,2_)* | -2.14 - 2.30 | -4.43 | -0.80 |
| **haplotype *I.II*** | *res_A_(x_1,1_) + res_B_(x_1,2_)* | 2.14 - 2.30 | -0.16 | -2.09 |
| **haplotype *II.I*** | *res_A_(x_1,2_) + res_B_(x_1,1_)* | -2.14 + 2.30 | 0.16 | -1.59 |
| **haplotype *I.I*** | *res_A_(x_2,1_) + res_B_(x_2,1_)* | - 2.14 - 2.30 | -4.43 | -3.31 |
| **haplotype *II.II*** | *res_A_(x_2,2_) + res_B_(x_2,2_)* | 2.14 + 2.30 | 4.43 | 0.80 |
| **haplotype *I.II*** | *res_A_(x_2,1_) + res_B_(x_2,2_)* | -2.14 + 2.30 | 0.16 | 2.09 |
| **haplotype *II.I*** | *res_A_(x_2,2_) + res_B_(x_2,1_)* | 2.14 - 2.30 | -0.16 | 1.59 |

*res_Y_(x_i,j_)* indicates the adjusted residual allele frequency of allele *i* in hybrid group *j* at marker Y. The shaded part is included in the table for completeness, but was excluded from the regression (see below). As a consequence of rounding to two digits, some allele indicators do not match their calculation. We used the unrounded values in the regression.

In the regression of adjusted residual haplotype counts against the allele indicator statistic, the goal was to investigate if at certain marker pairs the adjusted residual haplotype counts deviated from their expected values based on the allele indicator statistic. In that context, comparing hybrid groups *viable I* to *inviable I* is equivalent to comparing *inviable I* to *viable I*. This is reflected in the adjusted residual haplotype counts, as well as in the allele indicators, where half of the values are additive inverses of the other half. Consequently, for our purpose one of the two comparisons is redundant. To avoid pseudoreplication, we therefore included only (the non-shaded) half of the values in the regression.

**References in supporting information**

Boubou, A., Migeon, A., Roderick, G.K., Auger, P., Cornuet, J., Magalhães, S., and Navajas, M. (2012). Test of colonization scenarios reveals complex invasion history of the red tomato spider mite *Tetranychus evansi*. *PLoS One 7*: e35601.

Feng, H., Wang, L., Liu, Y., He, L., Li, M., Lu, W., and Xue, C. (2010). Molecular expression and characterization of a heat shock protein gene (HSP90) from the carmine spider mite, *Tetranychus cinnabarinus* (Boisduval). *Journal of Insect Science 10*: 1-14.

Fukatsu, T., Tsuchida, T., Nikoh, N., and Koga, R. (2001). *Spiroplasma* symbiont of the pea aphid, *Acyrthosiphon pisum* (Insecta: Homoptera). *Applied and Environmental Microbiology 67*: 1284-1291.

Klindworth, A., Pruesse, E., Schweer, T., Peplies, J., Quast, C., Horn, M., and Glöckner, F.O. (2013). Evaluation of general 16S ribosomal RNA gene PCR primers for classical and next-generation based diversity studies. *Nucleic Acids Research 41*: e1.

O’Neill, S.L., Giordano, R., Colbert, A.M.E., Karr, T.L., and Robertson, H.M. (1992). 16S rRNA phylogenetic analysis of the bacterial endosymbionts associated with cytoplasmic incompatibility in insects. *Proceedings of the National Academy of Sciences USA 89*: 2699-2702.

Weeks, A.R., Velten, R., and Stouthamer, R. (2003). Incidence of a new sex-ratio-distorting endosymbiotic bacterium among arthropods. *Proceedings of the Royal Society London B 270*: 1857-1865.
